# Severe Colchicine Intoxication: A Case Report and Review of Literature in Adults

**DOI:** 10.7759/cureus.19151

**Published:** 2021-10-30

**Authors:** Rui Seixas, Diogo Lopes, Marta Couto, José Pereira, José Paiva

**Affiliations:** 1 Internal Medicine, Unidade Local Saúde Litoral Alentejano, Santiago do Cacém, PRT; 2 Clinical Pharmacology Unit, Centro Hospitalar Universitário de São João, Porto, PRT; 3 Emergency and Intensive Care Department, Centro Hospitalar Universitário de São João, Porto, PRT; 4 Medicine Department, Faculty of Medicine of University of Porto, Porto, PRT; 5 Infection and Sepsis Group, Centro Hospitalar Universitário de São João, Porto, PRT

**Keywords:** mortality, therapy, intensive care unit, intoxication, colchicine, adults

## Abstract

Colchicine is used in the treatment of multiple diseases such as gout and auto-immune diseases. Although cases of multiorgan failure have been described in adults with doses usually higher than 0.8 mg/kg, the epidemiology data are scarce and the intoxication mechanisms are not well-known. The authors present the case of a 66-year-old male patient, with a medical history of depression, admitted to the emergency room (ER) due to intentional colchicine ingestion after taking 90 mg (approximately 1.125 mg/kg) 12 hours prior to medical evaluation. Besides some dizziness and sleepiness, he showed no other alteration in the physical examination. After the administration of activated charcoal, he was transferred to the intensive care unit (ICU). The laboratory findings showed mild hepatic dysfunction, acute kidney injury, and metabolic lactic acidosis. Despite treatment, severe clinical worsening with multiorgan failure, including respiratory failure complicated with multiple episodes of cardiac dysrhythmias and finally with persistent cardiac arrest, lead to the patient’s death after 13 hours of admission in the ER. Among drug intoxications, high doses of colchicine ingestion can lead to rapid multiorgan dysfunction, and patients with a severe overdose can experience irreversible multiorgan failure without presenting the typical initial gastrointestinal symptoms. Thus, it is necessary for the physicians to be alert to these situations and to be aware of the epidemiological data and clinical profile of this specific poisoning that should be managed in ICU. The authors perform a review of the cases of colchicine poisoning reported in adults between 2017 and 2019 and the differences in clinical management and outcomes.

## Introduction

Colchicine is a drug commonly used to treat gout or auto-immune diseases due to its anti-inflammatory properties. Physicians should be aware that though colchicine poisoning is not common, it can be life-threatening. The lethal dose is considered to be 0.8 mg/kg [1] but severe cases have been reported in adults with lower doses [2]. Here, we report a case of a patient who voluntarily ingested a high dose of colchicine in a suicide attempt with a fatal outcome. We review the literature about the subject, pharmacology of the drug, toxicological pathophysiology, and evidence about therapeutic support.

## Case presentation

A 66-year-old male patient, with a history of arterial hypertension, type 2 diabetes, chronic obstructive pulmonary disease, and depression, without medical follow-up was admitted to the emergency room (ER) after being found by his wife next to several empty packages of colchicine. According to the patient, he had voluntarily ingested 90 pills of colchicine (1.125 mg/kg) 12 hours prior to the medical evaluation. On arrival at the ER, his physical examination did not show any relevant findings besides mild dizziness. He was hemodynamically stable with blood pressure (BP) of 131/68 mmHg, pulse of 73 beats per minute (bpm), blood oxygen saturation (SpO2) of 98% on room air, and Glasgow Coma Scale (GCS) of 15 points. The patient denied abdominal pain, vomiting or diarrhea, or respiratory distress. His bloodwork, however, revealed liver and kidney dysfunction. The arterial blood gas analysis revealed metabolic acidosis and hyperlactacidemia - pH 7.47; bicarbonate (HCO3-) 19 mmol/L, and lactate 4.0 mmol/L. Laboratory results on admission are shown in Table 1.

**Table 1:** Laboratory Data on Admission

Laboratory Data	Value	Units	Value Range
White blood cells	24.140	x 10^9^/L	4.0 – 11.0
Hemoglobin	18.6	g/dL	13.0 – 18.0
Platelets	340	x 10^9^/L	150 – 400
Blood urea nitrogen	40	mg/dL	10 - 50
Creatinine	1.31	mg/dL	0.67 – 1.17
Aspartate aminotransferase	295	U/L	10 - 37
Alanine aminotransferase	84	U/L	10 - 37
Gamma-glutamyltransferase	75	U/L	10 - 49
Alkaline phosphatase	174	U/L	30 - 120
Lactate dehydrogenase	2039	U/L	135 - 225
Creatine kinase	214	U/L	10 - 172
Sodium	141	mEq/L	135 - 147
Potassium	2.9	mEq/L	3.5 - 5.1
C-reactive protein	27.4	mg/L	< 3.0
Activated partial thromboplastin time	30.2	seconds	24.2 - 36.4
Prothrombin time	17.8	seconds	9.9 - 13.6
Prothrombin and proconvertin	0.53	U/mL	0.70 - 1.25

Treatment with activated charcoal was initiated, and the patient was admitted to the intensive care unit (ICU). On arrival at the ICU, he showed increased sleepiness with a GCS of 14 points. Tympanic temperature was 37.6ºC. Due to his hepatic dysfunction, empirical treatment with high doses of acetylcysteine was started according to the hospital protocol for acetaminophen overdose (loading dose: 12000 mg in the first hour, 4000 mg over the next four hours, and finally 8000 mg over 16 hours). He began supplementation of oxygen through a Venturi mask with an inspired oxygen fraction of 31%. However, repeated blood gas analysis showed mild respiratory dysfunction with a partial pressure of oxygen (paO2)/fraction of inspired oxygen (FiO2) ratio of 225, worsening metabolic acidosis with HCO3- of 16,7 but maintaining a pH 7.44 due to hyperventilation. Lactate was 5.13 mmol/L. Five hours after admission, the patient presented fever (38.8ºC) with polypnea - respiratory rate (RR) of 30 to 40 breaths per minute) with worsened respiratory dysfunction, which led to intubation and invasive mechanical ventilation. He developed cardiac arrest (asystole) and was resuscitated after six minutes of advanced life support. Norepinephrine was started after recovery of spontaneous circulation, at 0.4 mcg/kg/min (maximum dosage given). Nine hours post-admission to the ICU, the patient developed anuria with worsening metabolic acidosis, hyperlactacidemia, and multiorgan dysfunction (respiratory, renal, hepatic, and hematologic), presenting a new episode of cardiac arrest with asystole reverted after six minutes. Renal replacement therapy with continuous venovenous hemofiltration (CVVH) was initiated. A cardiac ultrasound evaluation was performed, and it revealed severe biventricular dysfunction. Empirical antibiotherapy with amoxicillin and clavulanic acid was started due to progressive elevation of fever and elevated C-reactive protein even though no infectious cause was identified. Four hours later (13 hours post-admission in the ICU and 25 hours post-ingestion of colchicine), he again went into cardiac arrest (asystole) and advanced life support was unsuccessful. The progression of laboratory findings is shown in Table 2.

**Table 2:** Laboratory Data on Admission and Twelve Hours After Admission in the ICU

Laboratory Data	Hour 0 (admission)	Hour 12	Units
White blood cells	24.140	22.690	x 10^9^/L
Hemoglobin	18.6	15.60	g/dL
Platelets	340	152	x 10^9^/L
Creatinine	1.31	3.25	mg/dL
Creatine kinase	214	473	U/L
Aspartate aminotransferase	295	409	U/L
Alanine aminotransferase	84	125	U/L
Gamma-glutamyltransferase	75	76	U/L
Alkaline phosphatase	174	192	U/L
Lactate dehydrogenase	2039	3255	U/L
C-reactive protein	27.4	131.9	mg/L
Activated partial thromboplastin time	30.2	143.0	seconds
Prothrombin time	17.8	57.2	seconds
Prothrombin and proconvertin	0.53	0.13	U/mL

**Figure 1:**
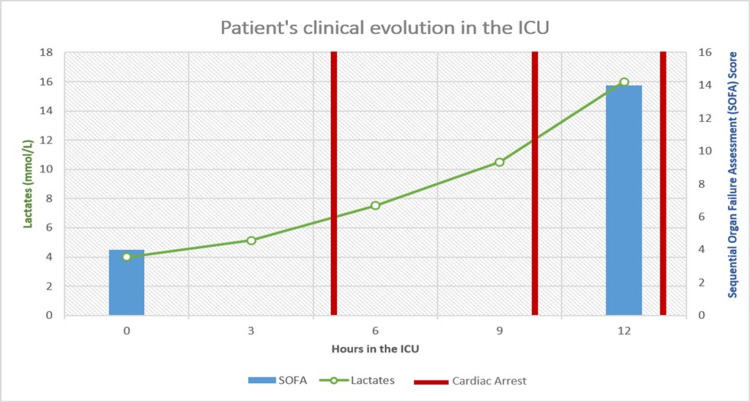
Post-Admission Clinical Evolution in the ICU

Figure 1 depicts the clinical progression of the patient over time in the ICU, showing the progressive elevation of the lactate level (minimum of 4 mmol/L and a maximum of 16 mmol/L), the increase in the sequential organ failure assessment (SOFA) score from four points to 14 points (predicting a ≥95.2% mortality) as well as the three episodes of cardiac arrest.

## Discussion

A 66-year-old man voluntarily ingested 90 mg of colchicine (1.125 mg/kg), a dosage clearly higher than the fatal dose of 0.8 mg/kg. In a matter of 13 hours, he developed multiorgan failure, without any of the gastrointestinal symptoms usually described in the first 24 hours of colchicine ingestion. After that, his clinical evolution was catastrophic, with rapid progression of multiorgan damage with respiratory, neurological, renal, and liver failure, as well as cardiac electrical instability, resulting in a fatal outcome 13 hours after ICU admission (25 hours after the colchicine ingestion). Colchicine is a neutral lipophilic alkaloid with rapid, yet highly variable, oral absorption, with a serum peak within the first three hours after ingestion. The compound has an extensive hepatic first-pass metabolism and a rapid distribution to all body tissues. The liver is the main route of elimination accounting for 80-90% of excretion [3-4]. Colchicine has been used for several years to treat several different inflammatory conditions with variable rates of success. It has a narrow therapeutic range that lies between 0.015 and 0.03 mg/kg [5] and even though that is generally safe in people without renal or liver failure, it is not uncommon for side effects to occur. For this reason, and due to the high risk of toxicity, intravenous formulations were withdrawn from the market [6]. The use of activated charcoal is usually reserved for an acute overdose of this drug (up to two hours after drug ingestion) since it adsorbs colchicine, preventing gastrointestinal absorption and interrupting its enterohepatic circulation [7]. Despite this, large amounts of colchicine have been found to remain in the stomach even 24 hours after ingestion [8] so, though this patient already had a late presentation of 12 hours, he was given activated charcoal upon admission. At a cellular level, colchicine exerts its properties mainly by interacting with microtubules, a polymerized structure composed of α and β subunits [9-10]. These structures have multiple functions inside the cell like transport, mitosis, cell signaling, gene expression, and motility. By binding to these subunits, colchicine interferes with microtubules assembly and, consequently, leads to the cell spindle disruption, cessation of mitosis, and impairment of main cell functions [6,10]. The mechanism by which toxicity and side effects occur may be extrapolated by their mechanism of action: disruption of the microtubules network, which results in altered cell shape, depressed cellular motility, arrest in metaphase, and decreased exocytosis and endocytosis [6]. This inhibition of cell division can affect all cells in the body, nevertheless, high cell-turnover body systems have a higher tendency to toxicity for obvious reasons. This fact explains why the bowel and hematopoietic system are particularly affected. It may also directly affect the heart through binding of the microtubules in cardiomyocytes, leading to impaired cardiac conduction and contractility, resulting in cardiac arrhythmias and cardiac failure [3,5-6]. Colchicine poisoning usually follows a three-stage model: the gastrointestinal phase, which appears on the first day after ingestion and presents with nausea, vomiting, abdominal pain, and bloody diarrhea. Shock may be present due to hypovolemia and heart failure. Then the second stage, characterized by multiorgan failure, which happens up to a week after toxic intake and is characterized by cardiac arrhythmias, liver failure, convulsions, pancytopenia, metabolic changes (metabolic acidosis, hypokalemia, hyponatremia, hypocalcemia, and hypophosphatemia), and neurologic syndrome with proximal limb weakness, distal sensory abnormalities, and neural conduction impairment. Death usually occurs during the first two days after intake by respiratory failure, intractable shock, cardiac arrhythmias, or sudden cardiac arrest. Finally, the recovery phase is characterized by organ recovery, alopecia, and rebound leukocytosis [4]. Chronic poisoning, although rare, can happen with neuromyopathy and myocardial failure in a more insidious way. However, in cases of massive ingestion, patients may evolve in a much faster and catastrophic way. Our patient appeared to skip entirely the first phase, not showing any signs of gastrointestinal distress and presenting with fully established distributive shock and multiorgan failure, a clinical evolution rarely described. Since cases of overdose with colchicine in adults are not frequent, there are not many case reports published (Table 3).

**Table 3:** Summary of Adult Cases With Colchicine Intoxication Between 2017 and 2019 NR: Not Reported; AC: Activated Charcoal; IMV: Invasive Mechanical Ventilation; FFP: Fresh Frozen Plasma; PRP: Platelet Rich Plasma; CRRT: Continuous Renal Replacement Therapy; NAC: N-Acetylcysteine; AB: Antibiotherapy; G-CSF: Granulocyte-Colony Stimulating Factor

Gender	Age	Cause of overdose	Dosage	Hours between ingestion and admission	Reported treatments	Outcome	Reference
Female	18	Pain relief	15 mg (0.2 mg/kg)	NR	ICU admission, IMV, blood transfusion, CRRT, G-CSF, AB	Recovery	Hirayama et al (2018) [2]
Female	18	Suicide (ingestion of Gloriosa superba)	NR	NR	ICU admission, IMV, CRRT, FFP, G-CSF	Fatal	Gunasekaran et al (2019) [11]
Female	52	Suicide	NR	NR	ICU admission, FFP	Fatal	Schreiber et al (2019) [12]
Female	19	Suicide	40mg	6	IMV, ICU admission, CRRT	Recovery	Zhong et al (2018) [13]
Female	18	Suicide	18 mg (~0.4 mg/kg)	72	ICU admission, AC, NAC, G-CSF, non-invasive mechanical ventilation	Recovery	Lev et al (2017) [14]
Male	70	Accidental (ingestion of Colchicum autumnale)	33ng/mL (Post-mortem)	Fatal prior to admission	Fatal prior to admission	Fatal	Giorgetti et al (2019) [15]
Female	NR	Accidental (ingestion of Colchicum autumnale)	32ng/mL	NR	CRRT	Fatal	Giorgetti et al (2019) [15]
Female	61	Drug–drug interaction (colchicine and clarithromycin)	2mg/d	NR	Hydration, colchicine suspension	Recovery	Yahia et al (2017) [16]
Female	36	Drug–drug interaction (colchicine and clarithromycin)	1.5mg	NR	NR	Recovery	Yahia et al (2017) [16]
Female	71	Drug–drug interaction (colchicine and clarithromycin)	1.5mg	NR	NR	Recovery	Yahia et al (2017) [16]
Female	41	Drug–drug interaction (colchicine and clarithromycin)	2.5mg	NR	NR	Recovery	Yahia et al (2017) [16]
Female	24	Drug–drug interaction (colchicine and clarithromycin)	2.5mg	NR	NR	Recovery	Yahia et al (2017) [16]
Female	69	Drug–drug interaction (colchicine and clarithromycin)	2mg	NR	Saline infusion, Colchicine suspension	Recovery	Yahia et al (2017) [16]

Most of the reported cases of colchicine intoxication are due to intent to self-harm [11-14], either by ingestion of colchicine-rich plants or several colchicine tablets. However, there are many other causes for colchicine intoxication such as accidental ingestion of colchicine-enriched plants or colchicine interaction with other drugs. Gunasekaran et al. describe the case of an 18-year-old female patient who intentionally ingested tubers of *Gloriosa superba*, an ornamental plant of the region of southeast Asia [11]. It is a toxic plant due to its high concentration of colchicine, often ingested for deliberate self-harm. The patient died despite having received ICU care with plasmapheresis, hemodialysis, and granulocyte-colony stimulating factor. In fact, several authors describe cases of colchicine overdose due to ingestion of colchicine-rich plants, the most common being *Colchicum autumnale*, a more ubiquitous variety, spreading across Europe and even New Zealand. Most cases reported due to consumption of this plant are due to accidental ingestion mistaken for saffron [15]. Drug interactions are also the main contributor to colchicine intoxication, especially clarithromycin. Yahia et al. [16] describe six cases of patients with daily doses of colchicine with overdose due to interaction with clarithromycin. All the patients had familial Mediterranean fever and were treated with colchicine; however, they were also being treated with clarithromycin due to *Helicobacter pylori* infection. Colchicine intoxication occurred despite intact kidney function but, as clarithromycin acts as a potent CYP3A4 inhibitor, it greatly increases the serum concentration of colchicine. Besides clarithromycin, other drugs have been reported to interact with colchicine such as cyclosporine [17]. Cyclosporine increases colchicine toxicity by inhibiting P-glycoprotein resulting in increased intracellular colchicine concentration and decreasing its hepatic and renal excretion of the drug. Cyclosporine also interacts with CYP3A4 decreasing the hepatic elimination of colchicine. Physicians should always take note of the patients’ concomitant medication, especially when dealing with older patients with impaired kidney function since it is a known risk factor for colchicine intoxication. Due to colchicine’s large volume of distribution and high protein binding, renal replacement therapy with hemodialysis or hemoperfusion will not remove it, but successful cases have been reported with the use of CVVH [18]. It is worth mentioning that our patient was treated with acetylcysteine since it may counteract the inhibiting effects of colchicine on endogenous antioxidants decreasing cell death, however, the exact mechanism is still not completely elucidated. Treatment with colchicine-specific Fab fragment antibodies has shown positive results in the management of colchicine overdose [19], but it was not used in our patient since it is not commercially available.

## Conclusions

Cases of massive ingestion of colchicine are rare, and cases with such a fulminant progression even more. This case is relevant since it shows that with ingestion of high doses of colchicine (higher than the fatal dose of 0.8 mg/kg) patients may present with a fulminant evolution of the multiorgan dysfunction even without showing signs of gastrointestinal distress (the typical first phase of colchicine overdose) with the need of rapid organ support and close follow-up. This patient showed swift and progressive multiorgan failure with electrical instability and died 13 hours post-admission in an intensive care unit (25 hours after colchicine ingestion). The authors raise the hypothesis that whether a more aggressive and precocious treatment with organ support should be initiated at an early stage, in order to prevent a full-blown shock with multiorgan failure, but further studies on the effects and treatment of massive overdose of this drug still are required.

## References

[1] Bismuth C, Gaultier M, Conso F (1977). Aplasie médullaire après intoxication aiguë à la colchicine. Nouv Presse Med.

[2] Hirayama I, Hiruma T, Ueda Y, Doi K, Morimura N (2018). A critically ill patient after a colchicine overdose below the lethal dose: a case report. J Med Case Rep.

[3] Finkelstein Y, Aks SE, Hutson JR (2010). Colchicine poisoning: the dark side of an ancient drug. Clin Toxicol (Phila).

[4] Brunton LL, Chabner BA, Knollmann BC. eds (2021). Goodman & Gilman's: The Pharmacological Basis of Therapeutics, 12e. https://accessmedicine.mhmedical.com/content.aspx?bookid=1613&sectionid=102124003.

[5] Stack J, Ryan J, McCarthy G (2015). Colchicine. New Insights to an Old Drug. Am J Ther.

[6] Leung YY, Yao Hui LL, Kraus VB (2015). Colchicine—update on mechanisms of action and therapeutic uses. Semin Arthritis Rheum.

[7] Zellner T, Prasa D, Färber E, Hoffmann-Walbeck P, Genser D, Eyer F (2019). The use of activated charcoal to treat intoxications. Dtsch Arztebl Int.

[8] Ellwood MG, Robb GH (1971). Self-poisoning with colchicine. Postgrad Med J.

[9] Ghawanmeh AA, Chong KF, Sarkar SM, Bakar MA, Othaman R, Khalid RM (2018). Colchicine prodrugs and codrugs: chemistry and bioactivities. Eur J Med Chem.

[10] Dubey KK, Kumar P, Labrou NE, Shukla P (2017). Biotherapeutic potential and mechanisms of action of colchicine. Crit Rev Biotechnol.

[11] Gunasekaran K, Mathew DE, Sudarsan TI, Iyyadurai R (2019). Fatal colchicine intoxication by ingestion of Gloriosa superba tubers. BMJ Case Rep.

[12] Schreiber L, Morovič M, Špacayová K, Halko R (2019). Colchicine extract suicidal lethal poisoning confirmation using high-resolution accurate mass spectrometry: a case study. J Forensic Sci.

[13] Zhong H, Zhong Z, Li H, Zhou T, Xie W (2018). A rare case report of heavy dose colchicine induced acute kidney injury. BMC Pharmacol Toxicol.

[14] Lev S, Snyder D, Azran C, Zolotarsky V, Dahan A (2017). Severe hypertriglyceridemia and colchicine intoxication following suicide attempt. Drug Des Devel Ther.

[15] Giorgetti A, Nalesso A, Cecchetto G, Pizzi M, Bellan A, Viel G, Montisci M (2019). Two fatal intoxications by colchicine taken for saffron. Clinical, medico-legal and forensic toxicological implications. Leg Med (Tokyo).

[16] Haj Yahia S, Ben Zvi I, Livneh A (2018). Colchicine intoxication in familial Mediterranean fever patients using clarithromycin for the treatment of Helicobacter pylori: a series of six patients. Rheumatol Int.

[17] Garrouste C, Philipponnet C, Kaysi S, Enache I, Tiple A, Heng AE (2012). Severe colchicine intoxication in a renal transplant recipient on cyclosporine. Transplant Proc.

[18] Rahman O, Jacobi J, Peters H, Mowry J, Sohail M (2018). Demonstration of colchicine clearance by continuous venovenous hemofilration (CVVH) in severe toxicity. Am J Crit Care Med.

[19] Baud FJ, Sabouraud A, Vicaut E (1995). Brief report: treatment of severe colchicine overdose with colchicine-specific Fab fragments. N Engl J Med.

